# Verletzungen der tibiofibularen Syndesmose: operative Technik

**DOI:** 10.1007/s00113-026-01746-3

**Published:** 2026-09-07

**Authors:** Stefan Rammelt, Christine Marx

**Affiliations:** UniversitätsCentrum für Orthopädie, Unfall- & Plastische Chirurgie, Universitätsklinikum Carl Gustav Carus an der TU Dresden, Fetscherstr. 74, 01307 Dresden, Deutschland

## Einleitung

Die Integrität des tibiofibularen Syndesmosenkomplexes ist von entscheidender Bedeutung für die Funktion von Sprunggelenk und Fuß. Verletzungen der Syndesmosenbänder sind häufig und können sowohl isoliert als auch im Rahmen von Frakturen des oberen Sprunggelenks auftreten. Sie entstehen in der Regel durch eine forcierte Außenrotation des Fußes gegen den Unterschenkel und gehen mit einer latenten oder manifesten Diastase der Knöchelgabel einher [1]. Häufig entstehen im Rahmen von Sprunggelenkfrakturen knöcherne Avulsionen an der Vorderkante von Tibia und Fibula sowie der Tibiahinterkante [2].

Ziele der operativen Therapie sind die anatomische Rekonstruktion der Tibiainzisur und die anatomische Einstellung der Fibula in dieselbe. Die verschiedenen Verfahren sollen im Folgenden dargestellt werden. Wichtigster Prognosefaktor nach instabilen Verletzungen der distalen tibiofibularen Syndesmose ist die anatomische, stabile Wiederherstellung der Knöchelgabel mit Einpassung der distalen Fibula in die Tibiainzisur. Die Reposition und knöcherne Fixierung von Syndesmosenausrissen stellen hierfür wichtige Instrumente dar.

## Indikation

**Abb. 1:**
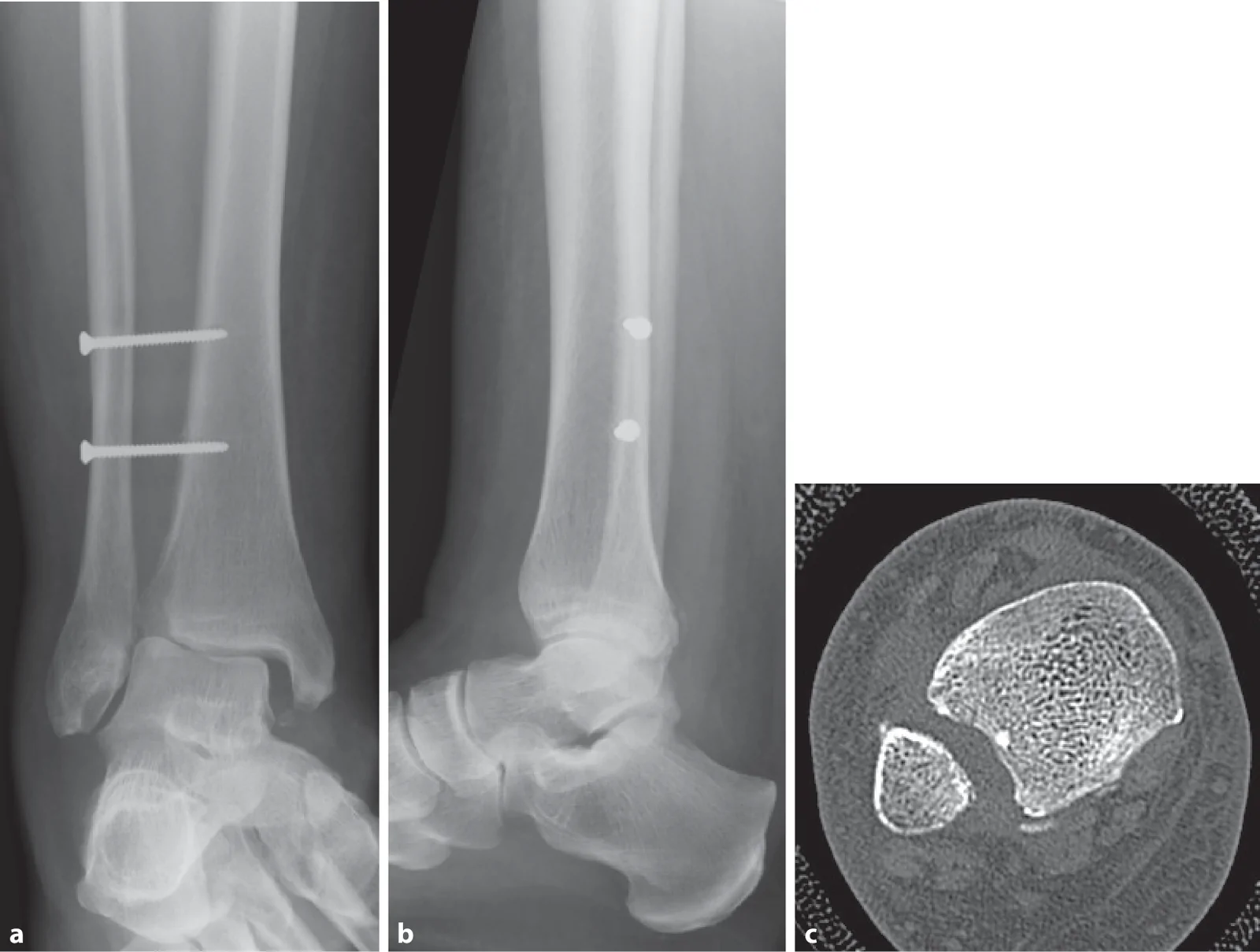
**a,b** Standardröntgenaufnahmen einer Patientin mit manifester Diastase nach perkutaner Stellschraubenfixierung einer instabilen, ligamentären Syndesmosenverletzung mit deutlich erweitertem Syndesmosenspalt (*tibiofibular clear space*, TCS), medialem Gelenkspalt (*medial clear space*, MCS) und Lateralversatz des Talus. **c** Der axiale CT-Schnitt zeigt die vordere Translation der distalen Fibula aus der Tibiainzisur mit nur minimaler, undislozierter Avulsion an der Tibiahinterkante und Fibulavorderkante.

Die Indikation zur operativen Therapie wird bei instabilen Syndesmosenverletzungen mit latenter oder manifester Diastase der Knöchelgabel und entsprechender Pathologie in der bildgebenden Diagnostik (tibiofibulare Distanz = TCS > 6 mm bzw. > 2 mm Seitendifferenz) gestellt (Abb. 1). Diese relevanten Syndesmoseninstabilitäten resultieren aus einer Ruptur von mindestens 2 Bandanteilen oder den entsprechenden knöchernen Avulsionsfrakturen [1, 3].

Die große Mehrzahl der Syndesmosenverletzungen entsteht im Rahmen von Sprunggelenkfrakturen. Die Wiederherstellung der Integrität und Stabilität des Syndesmosenkomplexes erfolgt in der Regel durch die Fixierung knöcherner Syndesmosenausrisse [2, 4]. Nach der Osteosynthese sämtlicher Frakturen und dislozierter knöcherner Avulsionen erfolgt die Stabilitätsprüfung der Knöchelgabel, zum Beispiel mittels Hakenzug nach dorsolateral oder Außenrotation des Fußes gegen die Tibia unter Durchleuchtung. Bei darstellbarer Diastase oder sagittaler Instabilität wird die distale Fibula in die Tibiainzisur reponiert und fixiert [5].

Für eine anatomische Reposition der distalen Fibula in die Tibiainzisur wird sowohl bei knöchernen als auch bei ligamentären Verletzungen das offene Vorgehen empfohlen, da es signifikant geringere Raten der prognostisch relevanten Fehlrepositionen aufweist [6].

## Isolierte ligamentäre Syndesmosenverletzungen

### Darstellung und Reposition

**Abb. 2:**
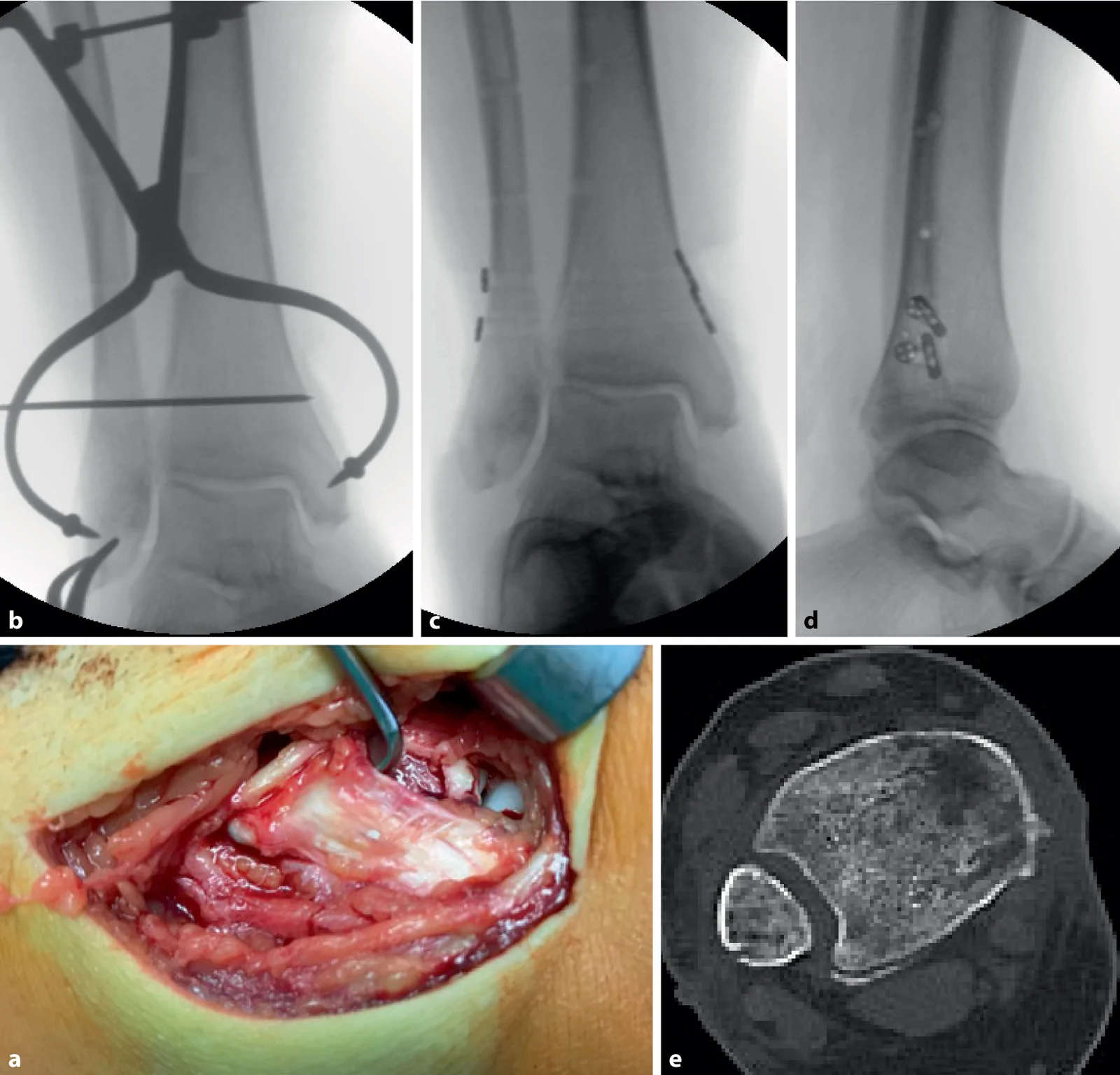
**a** Offene Reposition der distalen Fibula unter direkter Sicht auf das anterolaterale Gelenkkompartiment. Das vordere Syndesmosenband (AITFL) ist partiell von der Tibiavorderkante abgelöst. **b** Aufnahme mit aufgesetzter Repositionszange entlang der Achse des oberen Sprunggelenks und temporär eingebrachtem Kirschner-Draht. **c** Anteroposteriore und **d** seitliche Aufnahme nach Einbringen zweier leicht gegeneinander versetzter flexibler Implantate. **e** Die postoperative axiale CT zeigt die physiologische Einstellung der distalen Fibula in die Tibiainzisur. (Selbe Patientin wie in Abb. 1)

Die Syndesmosenregion wird über einen anterolateralen Zugang exploriert und von eingeschlagenen Kapsel- und Bandanteilen befreit (Abb. 2). Die laterale Taluskante wird auf chondrale oder osteochondrale Läsionen überprüft. Bei Verdacht auf medial gelegene Läsionen kann zusätzlich eine arthroskopische Exploration erfolgen. Unter manueller Tastkontrolle und direkter Sicht auf den lateralen Gelenkwinkel wird die distale Fibula in die Tibiainzisur reponiert und z. B. mit Hilfe einer geschwungenen Repositionszange über eine Stichinzision gegen den Malleolus medialis in reponierter Stellung gehalten. Die Rotation der Fibula stellt sich meist spontan ein, wenn der Fuß in Neutralstellung gehalten wird und kann, falls erforderlich, mit einem als Joystick eingebrachten Kirschner-Draht korrigiert werden [7].

Die Kontur des Tuberculum anterius fibulae sollte sich nach der Reposition in harmonischer Flucht an der Vorderkante des Tuberculum anterius tibiae fortsetzen [7, 8]. Unter direkter Sicht werden die Kongruenz und Parallelität der gelenkbildenden Flächen von Talus und Fibula im lateralen Winkel des oberen Sprunggelenkes kontrolliert [9, 10]. Die physiologische Position der Fibula ist in 97 % aller Fälle zentral oder in der anterioren Inzisur, eine Einstellung in der posterioren Inzisur legt eine Fehlreposition nahe [11]. Zur Erleichterung der Reposition ist es ratsam, den Unterschenkel auf einer Rolle zu lagern und den Fuß frei hängen zu lassen. Wird der Fuß bei durchhängendem Unterschenkel auf einer Rolle gelagert, kann es zu einer anterioren Translation der distalen Fibula durch die noch intakten Außenbänder kommen [12].

Die Repositionszange sollte vom zentralen Aspekt des Innenknöchels exakt in Sprunggelenkebene zur Fibulaspitze („*center-center*“) aufgesetzt werden, damit die Reposition der Fibula in beiden Ebenen in der physiologischen Achse erfolgt [1, 13, 14]. Die Zange soll dabei nur die Reposition halten und nicht durch übermäßigen Druck zu einer Überkompression führen [15]. Einige Autoren bevorzugen daher eine manuelle Reposition der Fibula zur Vermeidung einer Fehlreposition oder Überkompression. Ist eine zufriedenstellende Reposition erzielt, kann die Reposition durch einen temporär eingebrachten tibiofibularen Kirschner-Draht gesichert werden, um das Risiko eines Repositionsverlustes bei der definitiven Fixierung zu minimieren [1, 8].

### Beachtung der individuellen Morphologie der Tibiainzisur

Auch für die intraoperative Reposition hat sich zuletzt die Bedeutung der variablen Morphologie der Tibiainzisur gezeigt. Bei einer tiefen Inzisur besteht das Risiko der Überkompression, im Falle einer geneigten Inzisur das Risiko der Translation der Fibula nach anterior bzw. posterior [16]. Hilfreich kann die seitliche Bildwandlerdarstellung der gesunden kontralateralen Knöchelgabel zum intraoperativen Vergleich sein. Alternativ erfolgt eine intraoperative 3D-Kontrolle, die jedoch aus logistischen Gründen nicht überall zur Verfügung steht und die Lagerung des Patienten auf einem Karbontisch erfordert [17]. Auch ist sie – insbesondere in der Hand erfahrender Chirurgen – der zweidimensionalen Kontrolle mit korrekter Beachtung der klinischen und radiologischen Landmarken bezüglich des Repositionsergebnisses nicht überlegen [18, 19].

### Retention und Implantatwahl

Nach temporärer Kirschner-Draht-Fixierung erfolgt die definitive Retention mit tibiofibularen Stellschrauben oder flexiblen Implantaten. Prinzipiell werden 2 Stellschrauben bzw. Implantate verwendet, sofern bei isolierter Syndesmosenruptur keine weiteren Implantate eingebracht werden. Die ideale Lokalisation der Fixierung ist Gegenstand anhaltender Diskussionen und Untersuchungen. Tano et al. postulierten in einer anatomischen Kadaverstudie die Orientierung für die Schraubenposition am fibrokartilaginären Ansatz des Lig. tibiofibulare interosseum [20]. Dieser bildet eine Prominenz an der medialen Fibula. Die Schrauben werden von lateral nach medial 25–30° ansteigend und leicht divergierend eingebracht, was der physiologischen Sprunggelenkachse in der Horizontalebene entspricht [1, 21]. Bei korrekter Platzierung der Repositionszange kann der Bohrkanal somit parallel zu den Branchen der Repositionszange platziert werden. Der Bohrkanal sollte auf der lateralen Kante (Crista) der Fibula beginnen [22, 23]. Die erste Schraube (bzw. das erste Implantat) sollte in der Ideallinie (center-center) liegen, die zweite leicht divergieren, wobei es hierfür keine konkreten Vorgaben gibt.

Die Schrauben bzw. flexiblen Implantate werden 2–3 cm proximal des tibiotalaren Gelenkspaltes eingebracht [1, 7]. Bei einer weiter proximalen Einbringung besteht zum einen die Gefahr einer Fehlplatzierung des Bohrkanals, da die Tibiainzisur hier aufhört und der Bohrer auf die nunmehr konvexe laterale Kante der Tibia trifft und dort abgleiten kann. Zum anderen besteht bei proximaler Einbringung das Risiko eines Bowing und somit einer leichten Valgusabweichung der distalen Fibula. Da der Recessus des oberen Sprunggelenks bis zu 15 mm nach proximal reicht und die Crista fibulae bis 3 cm proximal der Gelenkebene exakt zentral liegt, ist eine Stellschraubenplatzierung zwischen 2 und 3 cm oberhalb des Tibiaplafonds als ideal anzusehen [23]. Durchmesser, Länge und Material der gewählten Stellschrauben haben hingegen keinen signifikanten Einfluss auf das klinische und radiologische Resultat [1, 8].

Alternativ zur Stellschraubenimplantation stehen flexible Implantate zur Verfügung. Die Reposition und Platzierung der Bohrkanäle erfolgen analog zur Stellschraubenimplantation, bei Verwendung zweier Implantate werden diese leicht divergierend angelegt [8]. Auch wenn durch die Flexibilität der Implantate kleinere Fehlrepositionen bzw. Fehlplatzierungen der Bohrkanäle ausgeglichen werden können, die flexiblen Implantate also gewisse „fehlerverzeihende“ Eigenschaften aufweisen [24, 25], dürfen sie keinesfalls als Repositionswerkzeug missverstanden werden.

In einigen prospektiven Studien werden verbesserte Outcome-Scores, insbesondere in der frühen postoperativen Phase, für Patienten mit flexiblem Implantat im Vergleich zu Stellschrauben gesehen [26], die Mehrzahl der Analysen ergab jedoch keinen relevanten Unterschied bei den funktionellen Ergebnissen [27, 28].

## Sprunggelenkfrakturen mit instabiler Syndesmose

Die große Mehrzahl der Syndesmosenverletzungen entsteht im Rahmen von Sprunggelenkfrakturen und wird entsprechend bei der operativen Versorgung instabiler Verletzungen über die hierfür verwendeten Zugänge adressiert [2, 29]. Verbleibt nach der Osteosynthese der Frakturen und Fixierung knöcherner Syndesmosenausrisse eine relevante Diastase oder sagittale Instabilität, wird die distale Fibula in die Tibiainzisur reponiert und fixiert [5]. Die Repositionstechnik entspricht dem Vorgehen bei instabiler, rein ligamentärer Syndesmosenverletzung. Zur Fixierung genügt jedoch eine Stellschraube bzw. flexibles Implantat.

**Abb. 3:**
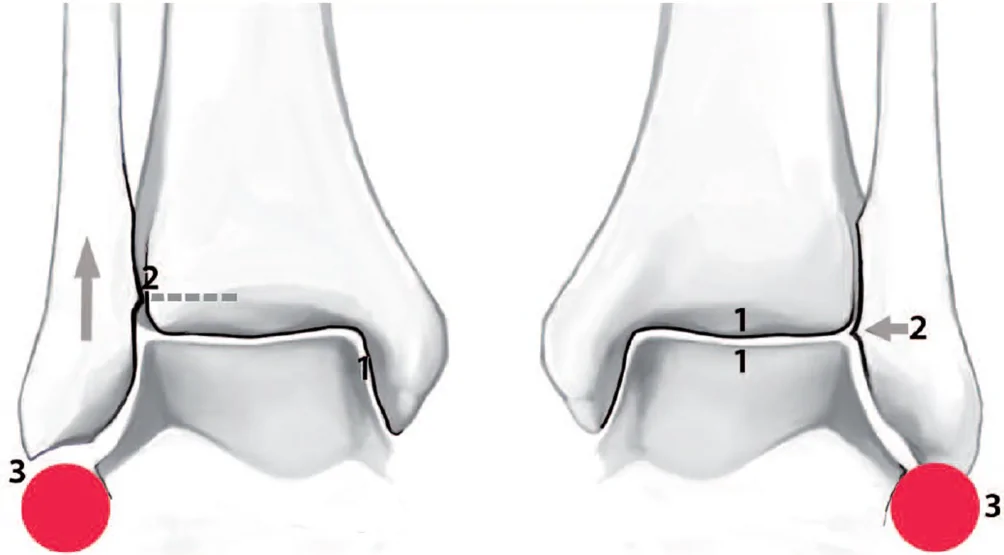
Weber-Indizes [30] zur Kontrolle der korrekten Reposition der Knöchelgabel: (*1*) Gleiche Weite von oberem und medialem Gelenkspalt, (*2*) Weber-Nase als prominenter Abschluss der medialen fibularen Gelenkfläche und (*3*) Weber-Kreis (*dime sign*). Einzelheiten im Text. (Aus Rammelt et al. [48])

Neben den oben genannten Indizes für die Syndesmosenstabilisierung gilt bei Frakturen der Wiederherstellung von anatomischer Länge und Rotation der Fibula besonderes Augenmerk. Orientierung geben dafür die sog. Weber-Indizes [30]: die Weber-Nase („Menard-Shenton-Linie“ des Sprunggelenks) als prominenter Abschluss der medialen fibularen Gelenkfläche stellt sich auf Höhe der distalen tibialen Gelenkfläche ein (Abb. 3). Der Weber-Kreis (*dime sign*) lässt sich gedanklich an die Konturen von Fibulaspitze und Processus lateralis tali anlegen. In der Regel genügt eine Stellschraube oder ein flexibles Implantat zur stabilen Fixierung der Fibula in der Tibiainzisur [5, 29].

### Maisonneuve-Fraktur

**Abb. 4:**
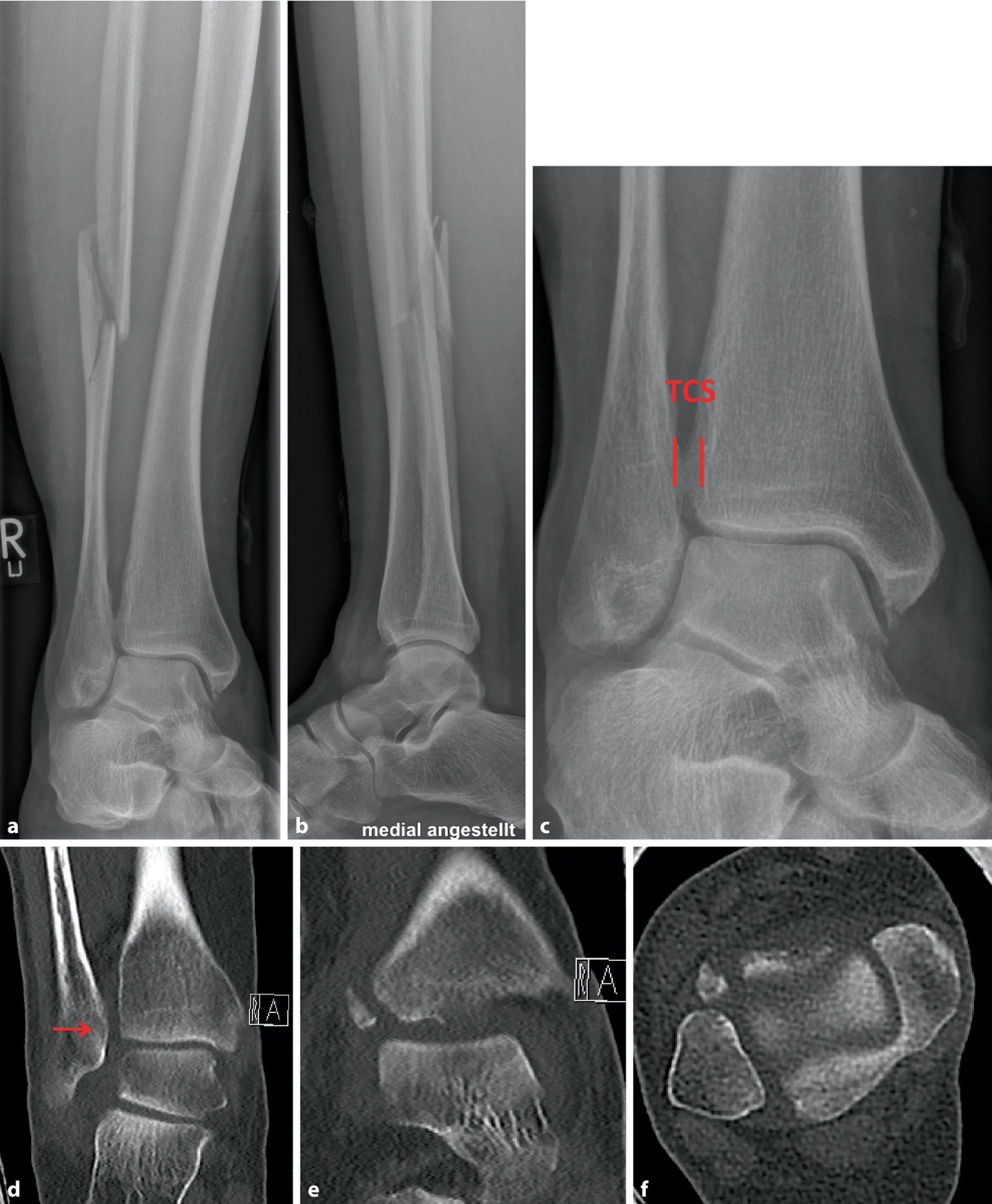
**a,b** Standardröntgenaufnahmen einer Patientin mit Maisonneuve-Verletzung und Verkürzung der Fibula. **c** Auf Höhe des oberen Sprunggelenkes imponiert ein erweiterter TCS bei normal erscheinendem MCS. **d** Die coronaren CT-Schnitte zeigt die Verkürzung der Fibula (*Pfeil* an der „Weber-Nase“) und **e** die nach lateral dislozierte Avulsionsfraktur des Tuberculum tibiale anterius (*Tubercule de Tillaux-Chaput*) mit Beteiligung der Gelenkfläche (Typ 2 [40]). **f** Im axialen CT-Schnitt zeigen sich zudem die Beteiligung der Fraktur an der Tibiainzisur und die rotatorische Komponente der Fehlstellung aufgrund der Syndesmoseninstabilität

Einen Sonderfall der Sprunggelenkfraktur mit meist instabiler Syndesmosenverletzung stellt die Maisonneuve-Fraktur dar (Abb. 4). Diese entsteht, wie auch die rein ligamentären Syndesmosenverletzungen, mutmaßlich durch forcierte Außenrotation bei proniertem Fuß [7]. Auf Höhe des oberen Sprunggelenks resultieren bei diesem Pathomechanismus eine Deltabandverletzung oder eine Innenknöchelfraktur sowie eine Syndesmosenruptur oder ein knöcherner Ausriss an der Tibiavorder- und/oder -Tibiahinterkante, mit Diastase oder Malrotation der distalen Fibula [31]. Die nach proximal fortgeleitete Gewalt produziert eine hohe Fibulafraktur oder Luxation des Fibulaköpfchens, wobei die Membrana interossea regelhaft nicht bis auf Höhe der proximalen Fibulaläsion rupturiert [32]. Liegt auf Höhe des oberen Sprunggelenks eine rein ligamentäre Verletzung vor, so erfolgt die Reposition der distalen Fibula in die Inzisur offen, wie oben angegeben, mit zusätzlichem Längszug. Eine Fixierung mit 2 Stellschrauben wird in diesen Fällen im eigenen Vorgehen bevorzugt, um auch eine ausreichende sagittale Stabilität zu gewährleisten [5]. Alternativ werden eine Stellschraube und ein flexibles Implantat in Hybridtechnik verwendet. Die Verwendung eines einzelnen flexiblen Implantats ist in diesen Fällen für die effektive tibiofibulare Stabilisierung in biomechanischen Versuchen nicht ausreichend [33, 34].

Nach operativer Versorgung einer instabilen Syndesmosenverletzung mittels Stellschraube oder flexiblem Implantat ist die CT-Kontrolle beider oberer Sprunggelenke obligat, sollte nicht bereits intraoperativ eine 3D-Bildgebung erfolgt sein [1, 8, 17]. Hierdurch kann die anatomische Reposition der Fibula in der Tibiainzisur im Seitenvergleich unter Beachtung der individuellen Morphologie überprüft werden.

### Knöcherne Syndesmosenausrisse

**Abb. 5:**
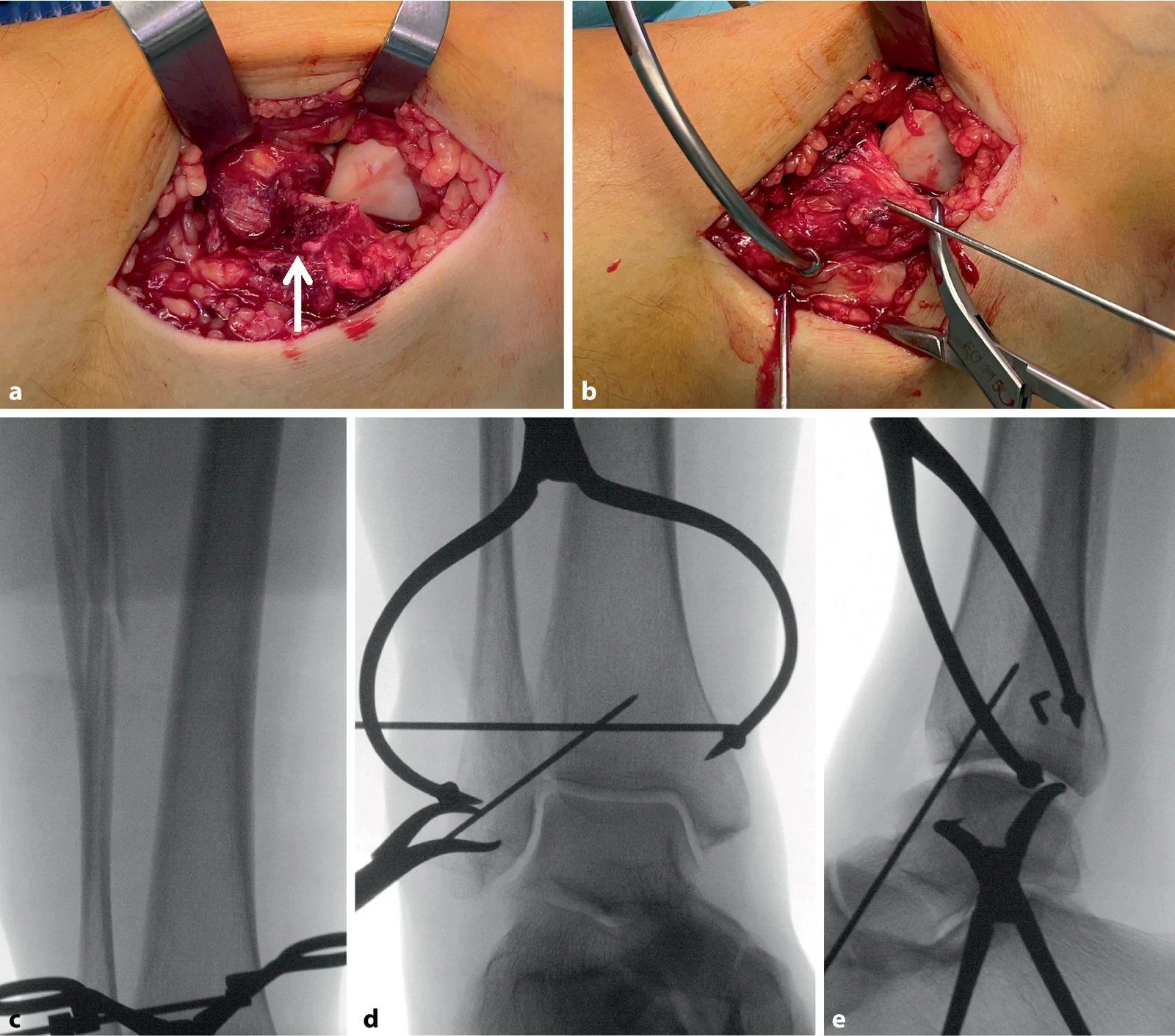
Intraoperative Aspekte mit Darstellung des dislozierten *Tubercule de Tillaux-Chaput* (*Pfeil* in **a**) sowie nach Reposition der distalen Fibula unter Längszug und mittels übergreifender Repositionszange (**b**). Anschließend gelingt die Einpassung des Tibiafragmentes mit dem AITFL. **c** Die hohe Fibulafraktur muss nicht direkt fixiert werden, die korrekte Länge wird jedoch im Bildwandler kontrolliert. **d** Im anteroposterioren und **e** im seitlichen Strahlengang wird Reposition nach den in Abb. 2 und 3 genannten Kriterien kontrolliert. (Selbe Patientin wie in Abb. 4)

Zeigt sich in der präoperativ durchgeführten Bildgebung ein dislozierter knöcherner Ausriss der vorderen oder hinteren Syndesmose, empfehlen sich die Exploration, anatomische Reposition und Refixierung (Abb. 5). Um eine möglichst hohe Stabilität zu erreichen, sollten alle Verletzungskomponenten adressiert werden [2, 5]. Die direkte Reposition und Stabilisierung von Syndesmosenavulsionen erlauben eine anatomische Wiederherstellung der Integrität der Syndesmose und knöcherne Stabilisierung der Fragmente, was der indirekten Stabilisierung und Platzierung von Stellschrauben bzw. flexiblen Implantaten biomechanisch überlegen ist [5, 35, 36]. In Abhängigkeit von der Lage und Größe der Fragmente werden auch die Kongruenz der Gelenkfläche zum Talus (Tibiaplafond) und die Integrität der Tibiainzisur wiederhergestellt, was die Reposition der distalen Fibula erleichtert und der Entwicklung einer posttraumatischen Arthrose vorbeugt [37, 38]. Da die kräftigen Tubercula anterius et posterius der distalen Tibia darüber hinaus einer Subluxation des Talus nach vorn bzw. hinten entgegenstehen, werden sie als „dritter“ bzw. „vierter“ Knöchel bezeichnet [5, 37] und Frakturen mit der Verletzung aller 4 knöchernen Komponenten als Quadrimalleolarfrakturen [39].

**Abb. 6:**
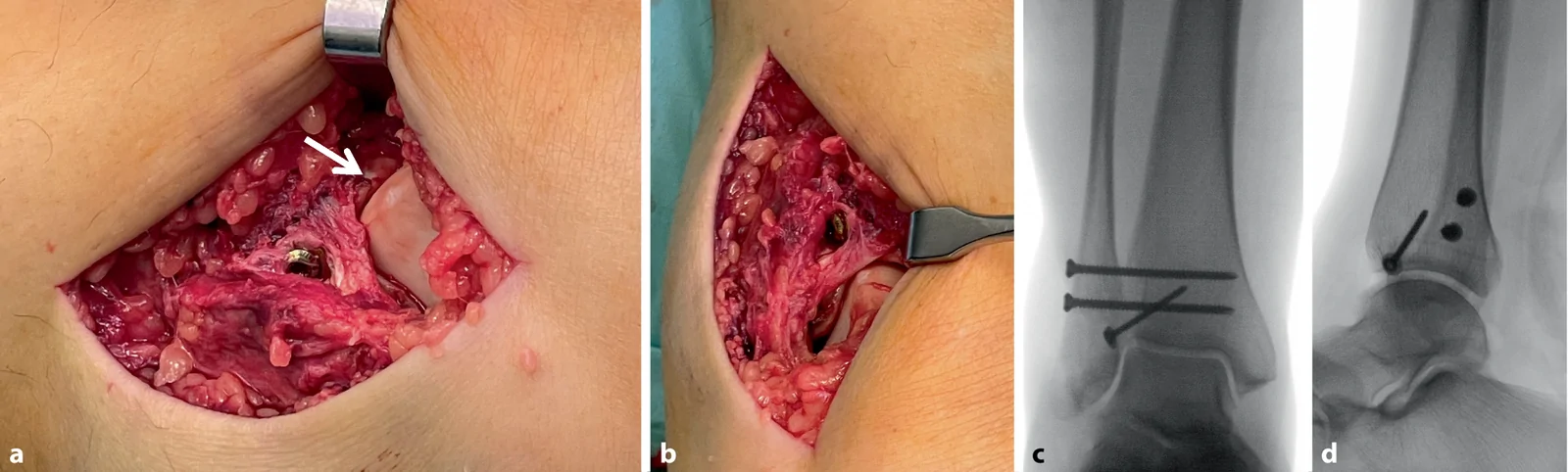
**a–d** Nach Verifizierung des korrekten Repositionsergebnisses werden 2 Stellschrauben implantiert. Das *Tubercule de Tillaux-Chaput* wird mit einer 2,7-mm-Schraube und Unterlegscheibe fixiert. Die Reposition der Gelenkfläche im anterolateralen Tibiaplafind wird unter direkter Sicht kontrolliert (*Pfeil* in **a**), die Rotation der Fibula durch die parallele Ausrichtung der Gelenkfläche an der lateralen Taluskante (**b**).  (Selbe Patientin wie in Abb. 4 und 5)

Ein dislozierter knöcherner Ausriss des Lig. tibiofibulare anterius an der distalen Tibia (*Tubercule de Tillaux-Chaput*) zeigt oft ein eingeschlagenes Periost im Frakturbereich, welches zunächst entfernt und der Bruchspalt gesäubert werden muss. Anschließend lässt sich das Fragment anatomisch einpassen und im Falle einer reinen Avulsionsverletzung (Typ 1 nach Rammelt et al. [40]) zumeist mittels Nahtanker fixieren. Dislozierte größere Fragmente mit Beteiligung der Gelenkfläche und der Inzisur (Typ 2) sollten zur Vermeidung einer posttraumatischen Arthrose offen reponiert und mit einer 2,7- bis 3,5-mm-Schraube mit Unterlegscheibe oder Miniplatte je nach Fragmentgröße, fixiert werden (Abb. 6). Impressionen der anterolateralen Gelenkfläche (Typ 3) erfordern die Defektanhebung und Unterfütterung mit Spongiosa und eine anterolaterale Antigleitplatte [37]. Das Vorgehen bei disloziertem Wagstaffe-Fragment an der ventralen Fibula (Quadrimalleolaräquivalent) ist analog [2, 41].

Dislozierte Frakturen der Tibiahinterkante werden je nach individueller Pathoanatomie fixiert. Posterolaterale Avulsionsfrakturen (Typ 2 nach Bartoníček et al. [42]) werden im eigenen Vorgehen in Bauchlagerung des Patienten über einen posterolateralen Zugang im ersten Schritt des Eingriffs adressiert. Zweiteilefrakturen mit Ausdehnung in den Innenknöchel (Colliculus posterior, Typ 3) können über einen posterolateralen oder posteromedialen Zugang direkt reponiert und fixiert werden [36]. Die direkte Reposition und Retention mittels Kleinfragmentschraube oder Antigleitplatte vor der Osteosynthese der Fibula- und/oder Innenknöchelfraktur ermöglicht die überlagerungsfreie Röntgenkontrolle der Tibiahinterkante und trägt bereits zur Reposition der distalen Fibula bei [2]. Bei großen triangulären Fragmenten (Typ 4) können alternativ eine indirekte Reposition und a.-p.-Zugschraubenosteosynthese erfolgen, wenn eine ausreichende Sicht auf die Tibiafraktur (z. B. durch eine Aufspreizung der Fibulafraktur) gewährleistet ist [36].

**Abb. 7:**
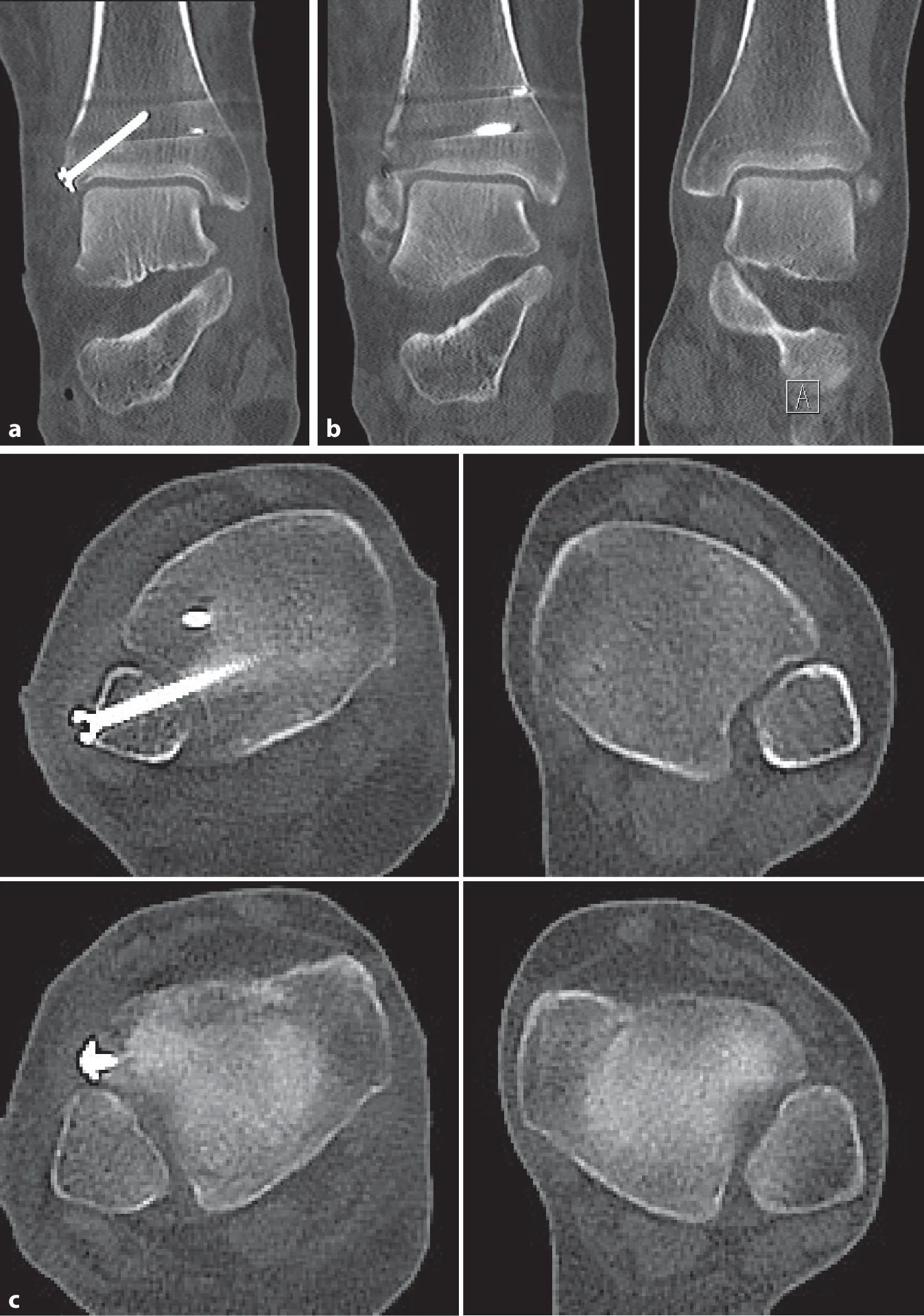
**a** Die postoperative CT-Kontrolle zeigt die Wiederherstellung der Gelenkfläche durch die Refixierung des *Tubercule de Tillaux-Chaput* sowie **b** die harmonische, seitengleiche Stellung der Knöchelgabel. **c** Die axialen CT-Schnitte demonstrieren die Wiederherstellung der Tibiainzisur und korrekte Einstellung der distalen Fibula in diese im Seitenvergleich. (Selbe Patientin wie in Abb. 4, 5 und 6)

Insbesondere für die Tibiahinterkante konnte in klinischen und biomechanischen Versuchen gezeigt werden, dass die Refixierung selbst kleinerer knöcherner Syndesmosenausrisse wesentlich zur Stabilität der Knöchelgabel beiträgt [35, 36, 43]. Außerdem stellt die anatomische Refixierung größerer knöcherner Avulsionsfrakturen an der anterioren und posterioren Tibia neben der Gelenkfläche auch die Integrität der Inzisur wieder her (Abb. 7), was wiederum die korrekte Reposition der Fibula erleichtert [2, 37].

### Nachbehandlung

**Abb. 8:**
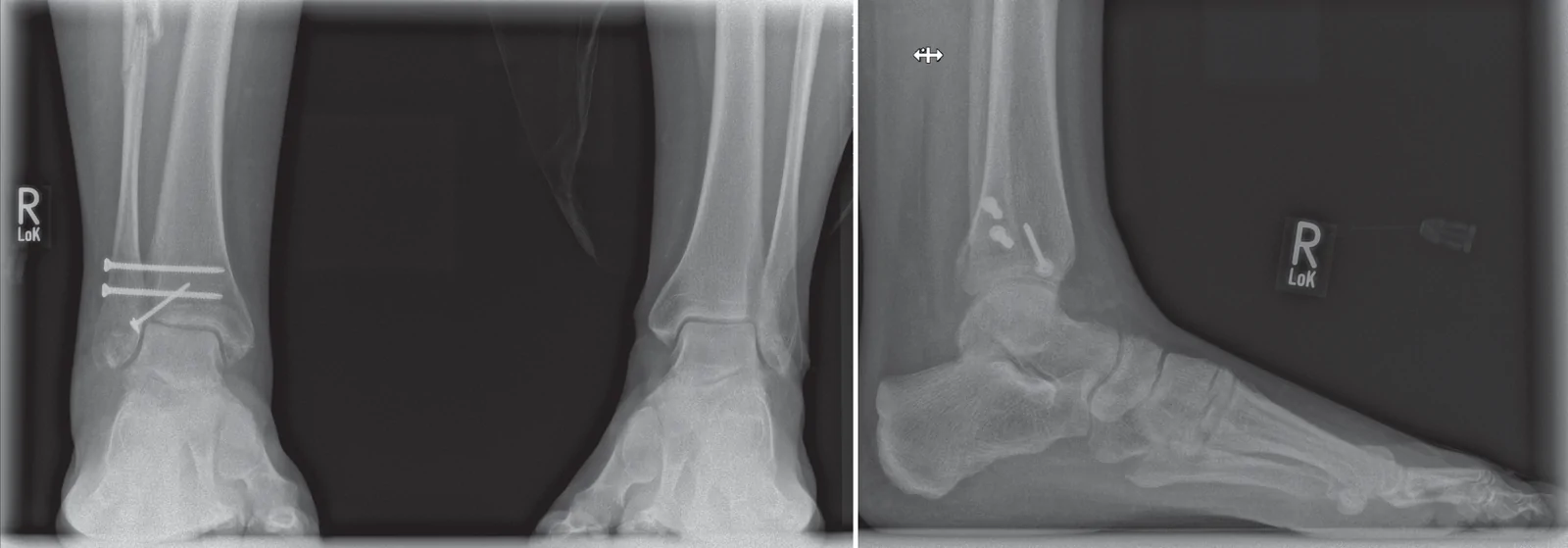
Die Röntgenkontrolle im Stand nach 6 Wochen zeigt die harmonische, seitengleiche Stellung der Knöchelgabel und somit stabile Ausheilung. (Selbe Patientin wie in Abb. 4, 5, 6 und 7)

Direkt postoperativ erfolgen die Hochlagerung und Ruhigstellung im Spezialstiefel, in einer Schiene oder einem gespaltenen Cast-Verband, abhängig vom lokalen Weichteilbefund. Bei reizlosen Wundverhältnissen kann nach zwei Tagen mit der Beübung der Sprunggelenke begonnen werden, um ein gutes funktionelles Ergebnis zu erreichen. Nach operativ versorgten Sprunggelenkfrakturen mit direkter Refixierung knöcherner Syndesmosenausrisse ist die beschwerdeadaptierte Vollbelastung im eigenen Schuh oder in einer Orthese möglich [44]. Abhängig von der Frakturmorphologie (vor allem bei Impaktierung des Tibiaplafonds) und der Knochenqualität erfolgt nach Einbringen von Stellschrauben bzw. flexiblem Implantat die Mobilisation zunächst unter Teilbelastung. Im Regelfall sind 6 Wochen mit 20 kg ausreichend. Nach abschließender Röntgenkontrolle ist die Belastungssteigerung möglich (Abb. 8). Eine aktuelle Studie fand jedoch auch nach indirekter Fixierung der Syndesmose keine erhöhte Komplikationsrate bei früher, beschwerdeadaptierter Vollbelastung [45].

Eine Entfernung der Stellschraube(n) ist nur bei symptomatischen Patienten mit persistierendem Engegefühl und Bewegungseinschränkung erforderlich, was in etwa 20 % innerhalb von 2 Jahren der Fall ist [46]. Auch bei flexiblen Implantaten kann eine Entfernung bei lokaler Weichteilirritation, insbesondere medial, in bis zu 18 % der Fälle notwendig sein [47].

## Fazit für die Praxis

- Ziele der operativen Versorgung von ligamentären und knöchernen Syndesmosenverletzungen sind die anatomische Reposition und stabile Fixierung der distalen Fibula in die Tibiainzisur mit Wiederherstellung der physiologischen Integrität der Knöchelgabel.
- Um eine anatomische Reposition der Knöchelgabel zu erreichen und zu beurteilen, muss die individuelle Morphologie der Tibiainzisur berücksichtigt werden.
- Prognoserelevant ist die anatomische Reposition der distalen Fibula in die Tibiainzisur, unabhängig von der Wahl des Implantates. Trotz eines gewissen fehlerverzeihenden Charakters sollten flexible Implantate nicht als Repositionsinstrument fehlverstanden werden.
- Die direkte Refixierung knöcherner Syndesmosenausrisse rekonstruiert die Tibiainzisur, erleichtert die Reposition der distalen Fibula und bietet eine höhere Stabilität und Repositionsqualität als die indirekte Fixierung des Syndesmosenkomplexes mittels Stellschraube oder flexiblem Implantat. Die Nachbehandlung erfolgt frühfunktionell mit aktiven und passiven Bewegungsübungen sowie unter beschwerdeadaptierter Vollbelastung nach direkter und etwas vorsichtiger nach indirekter Syndesmosenstabilisierung.

## Data Availability

Alle hier enthalten
